# Supplementary material for: Durable Janus membrane with on-demand mode switching fabricated by femtosecond laser
Source: Nat Commun. 2024 Feb 16;15:1443. doi: 10.1038/s41467-024-45926-4 (PMC10873403; doi:10.1038/s41467-024-45926-4)
Supplement: Supplementary file 1 — Supplementary Information [file 41467_2024_45926_MOESM1_ESM.pdf]

Supplementary Information

Durable Janus membrane with on-demand mode switching fabricated by femtosecond laser

*Zehang Cui[1,2,#], Yachao Zhang[1,3,#], Zhicheng Zhang[1], Bingrui Liu[4], Yiyu Chen[1,2], Hao Wu[1], Yuxuan Zhang[1], Zilong Cheng[1], Guoqiang Li[2], Jiale Yong[1], Jiawen Li[1], Dong Wu[1], Jiaru Chu[1] and Yanlei Hu[1]**

[1]CAS Key Laboratory of Mechanical Behavior and Design of Materials, Key Laboratory of Precision Scientific Instrumentation of Anhui Higher Education Institutes, Department of Precision Machinery and Precision Instrumentation, University of Science and Technology of China, Hefei 230027, China.

[2]School of Manufacture Science and Engineering, Key Laboratory of Testing Technology for Manufacturing Process, Ministry of Education, Southwest University of Science and Technology, Mianyang 621010, China.

[3]Anhui Province Key Laboratory of Measuring Theory and Precision Instrument, School of Instrument Science and Optoelectronics Engineering, Hefei University of Technology, Hefei 230009, China.

[4]Key Laboratory of Agri-Food Safety of Anhui Province, School of Resources and Environment, Anhui Agricultural University, Hefei, Anhui, 230036, China.

[#]These authors contributed equally: Zehang Cui, Yachao Zhang.

*Corresponding author. E-mail: huyl@ustc.edu.cn (Y.H.).

This file includes:

Supplementary Note 1

Supplementary Figures 1-18

Supplementary References

Note S1 Force analysis of water flow in a single microgroove

The capillary force of the liquid flow in microgrooves can be calculated from the free energy change. Here, a single microgroove on the surface is simplified to be rectangular with a width w and depth h (Supplementary Fig. 15c). The capillary force F_c is obtained as the negative rate of change in free energy with respect to the water flow filled grooves length x[1-3]:

$$F_c = -\frac{dE}{dx} = \gamma[(\cos\theta - 1)w + 2h\cos\theta] \tag{S1}$$

where E, γ and θ are the free energy change, water surface tension and water contact angle, respectively. For the water on hydrophilic PG channels surface $\cos\theta \approx 1$, that is:

$$F_c \approx 2\gamma h\cos\theta \tag{S2}$$

the values of groove depth before and after 2000 abrasion cycles are 82 and 43 μm, respectively. Hence, F_{c1} and F_{c2} are calculated to be 1.17×10^{-5} N and 6.16×10^{-6} N, respectively.

The viscous force F_v can be described as[4,5]:

$$F_v = \frac{3\eta x u}{\varepsilon\zeta(\varepsilon)} \tag{S3}$$

where η is the water viscosity, x is the water transport distance inside the microgroove, u is the flow velocity, the aspect ratio of the microgrooves is $\varepsilon = h/w$, and $\frac{1}{\zeta(\varepsilon)} = 1+0.671004\varepsilon+4.169711\varepsilon^2$. ε before and after 2000 abrasion cycles are about 1.41 and 0.74, the values of F_{v1} and F_{v2} are calculated to be 1.09×10^{-7} N and 4.91×10^{-8} N, respectively.

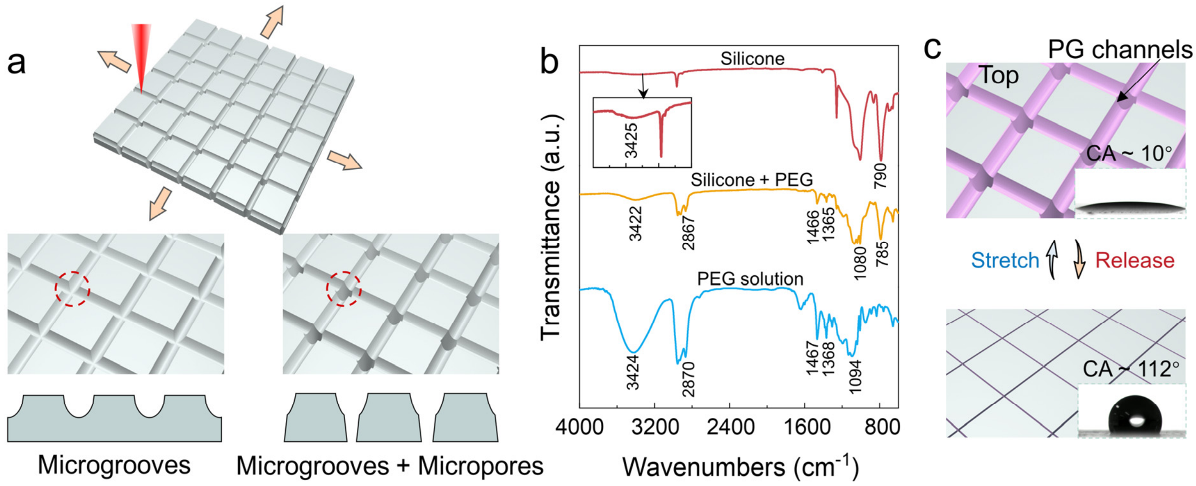

Fig. S1 Schematic illustration of hydrophilic PG channels fabrication process. **a** Microgrooves and micropores are fabricated on the pre-stretched membrane sequentially by femtosecond laser scanning and drilling. **b** Fourier transform infrared (FTIR) spectra of silicone, hydrophilic modified silicone and PEG solution. **c** Hydrophilized modification and wettability characterizations of the obtained PG channels in the two modes. Source data are provided as a Source Data file.

In the spectra of the silicone, peaks at 3425 and 790 cm^{-1} are assigned to the Si-OH and Si-O-Si, respectively. In the spectra of the hydrophilic reagent, the peaks at 3424, 2870 and 1094 cm^{-1} indicate the -OH, -CH_2O- and C-O-C bonds of PEG, respectively. In the spectra of the hydrophilic modified silicone membrane, the characteristic absorption peaks of PEG at 3424, 2870, 1467, 1368 and 1094 cm^{-1} also appear, which confirms that the PEG is incorporated with the silicone substrate. Compared with PEG solution, the peak at 3424 cm^{-1}, caused by the asymmetric stretching vibration of the -OH functional group, has a slight shift to 3422 cm^{-1} in hydrophilic silicone, which can be attributed to the intermolecular hydrogen bond interactions between the Si-OH of silicone and the terminal hydroxyl group of PEG. Caused by symmetric stretching vibration of the C-O-C functional group, the peak from 800 cm^{-1} to 1500 cm^{-1} of PEG also shifts to smaller wave number and the intensity weakens slightly, indicates the hydrogen bond interaction established between the Si-OH of silicone and the oxygen atom of C-O-C. The results show that no obvious new peaks are observed in the spectrum of hydrophilic silicone, indicating that there is physical absorption between the silicone substrate and the hydrophilic reagent[6].

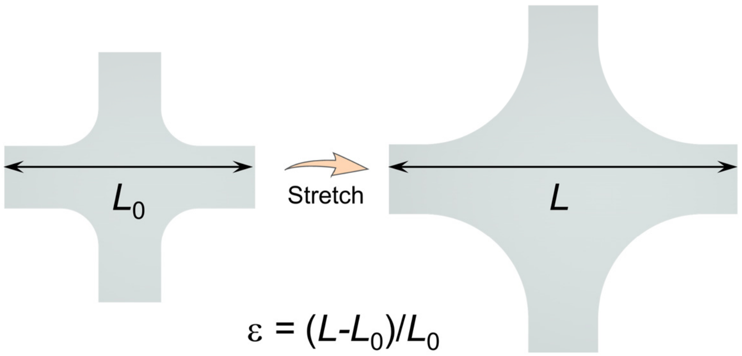

Fig. S2 Schematic diagram of the definition of stretching strain value ε.

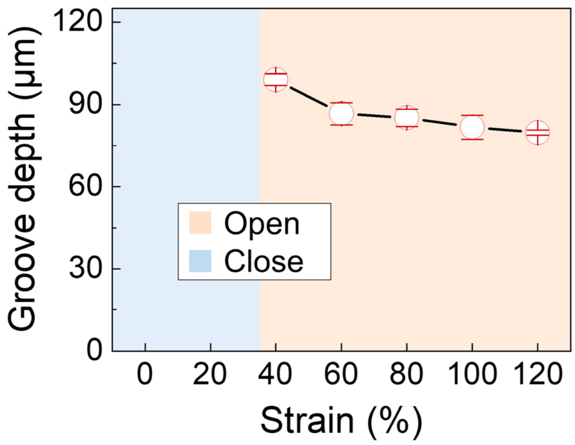

Fig. S3 Variation of groove depth under different strains. The depth changes to be immeasurable due to the closure of the groove at ε = 20% and 0. The error bars represent the standard deviation of three independent measurements. Source data are provided as a Source Data file.

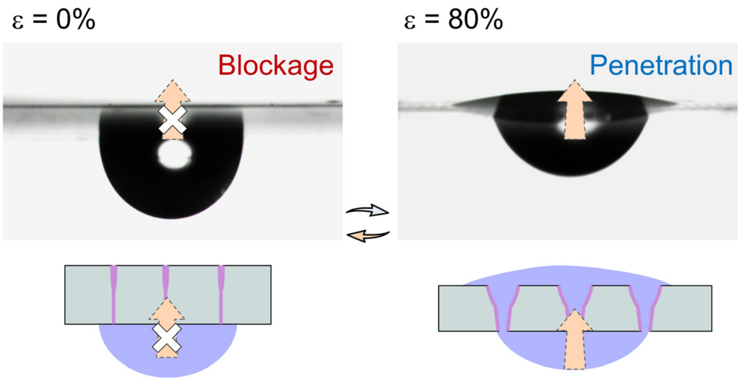

Fig. S4 Water unidirectional transport behaviors of blockage in protection mode and penetration in Janus mode, respectively.

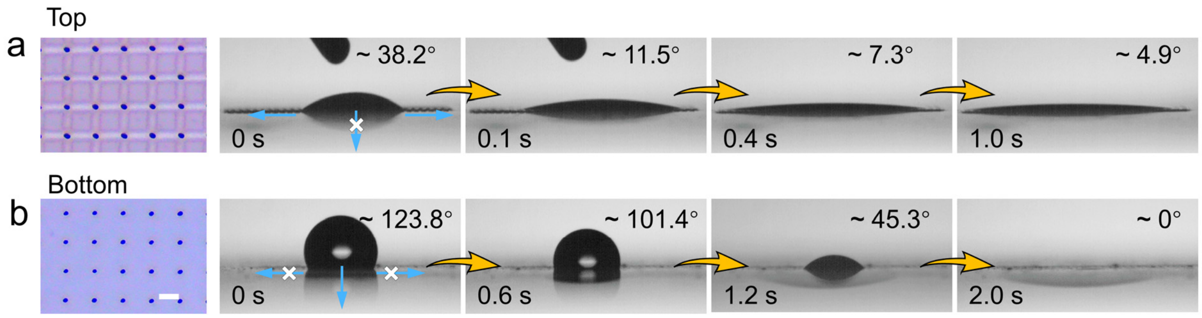

Fig. S5 Dynamic apparent contact angles snapshots of water dropped on **a** the top and **b** the bottom side of Janus membrane, respectively. Scale bar: 100 μm.

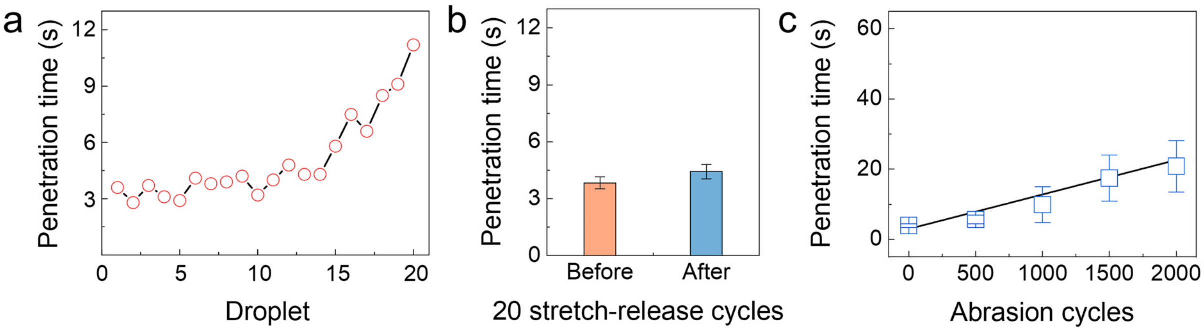

Fig. S6 Water unidirectional anti-gravity penetration time variations. **a** Time variation with continuously adding droplets in stretching Janus mode, **b** Time comparison for the first droplet on the original Janus membrane and on the Janus membrane after 20 stretch-release cycles, and **c** Time variation of a membrane sample with increasing abrasion cycles from 0 to 2000 cycles. The error bars represent the standard deviation of three independent measurements. Source data are provided as a Source Data file.

The unidirectional penetration time of the 20 droplets increases from 3.8 to 11.2 s in stretching Janus mode. The time for the first droplet before and after 20 stretching-releasing cycles increases slightly from 3.8 to 4.4 s. It indicates that the increase in penetration time with repeated cycles (Fig. 3g) is mainly due to the consumption of hydrophilic coating by the successively added droplets, rather than the repeated stretching and releasing of the membrane. The unidirectional penetration time of a sample increases from 3.8 s at 0 abrasion cycles to 20 s at 2000 abrasion cycles, compared with Fig. 4e, the excess time increasing is attributed to the consumption of hydrophilic coating and the reduced driving force caused by the mechanical abrasion.

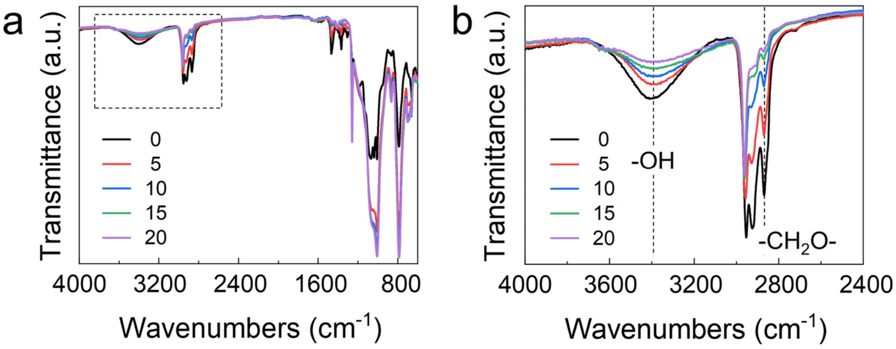

Fig. S7 a Fourier transform infrared (FTIR) spectrum of the hydrophilic silicone with during continuously adding droplets. **b** High-resolution FTIR spectrum of the dotted box in **a**. Source data are provided as a Source Data file.

The FTIR spectra are recorded for the 0th, 5th, 10th, 15th and 20th droplets. With the continuous addition of droplets from 0 to the 20th drop, the intensity of the characteristic peaks at 3422 and 2867 cm^{-1} (corresponding to the -OH and $-CH_2O$ of PEG) progressively decreases, which indicates that the decreasing PEG content on the silicone substrate, namely, the consumption of the hydrophilic coating.

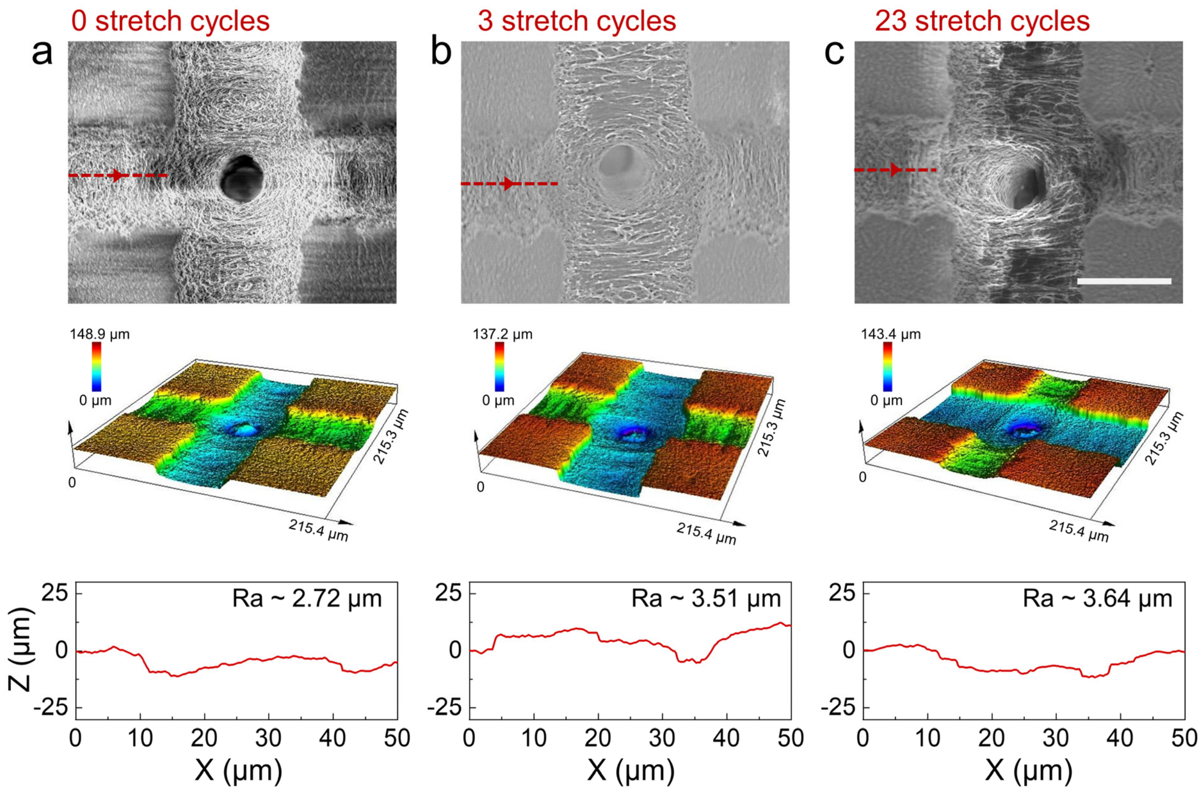

Fig. S8 SEM images, CLSM images and profile comparison of microgrooves morphology after different stretching and releasing cycles. Scale bar: 50 μm. Source data are provided as a Source Data file.

As the number of stretching cycles increases from 0 to 3, the surface roughness *Ra* increases from ~ 2.72 to 3.51 μm, which is attributed to the generation of cracks. Moreover, *Ra* remains constant as the number of stretching cycles increases from 3 to 23, which suggests that no new cracks appear. Three membrane samples after 0, 3 and 23 cyclic stretching/releasing with the same processing parameters are employed for SEM characterization.

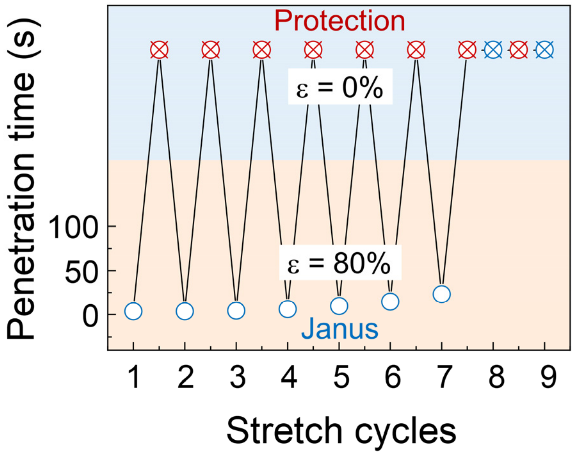

Fig. S9 Water unidirectional penetration of a Janus membrane with a single-time hydrophilic modification during repeated stretching and releasing cycles. Source data are provided as a Source Data file.

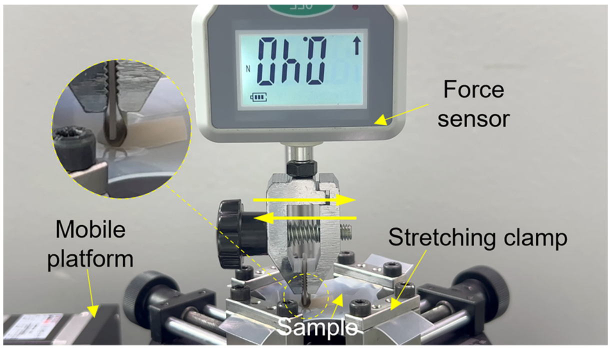

Fig. S10 Photograph of the mechanical long-term abrasion setup, with manometer reads upside down.

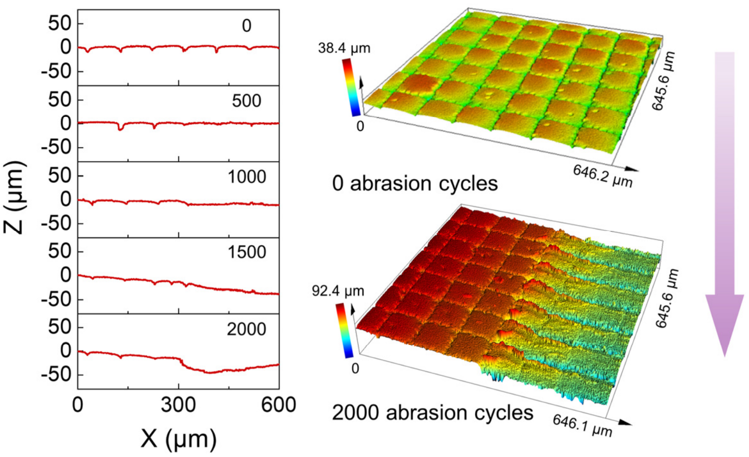

Fig. S11 Morphology and profile of the abrasion area under different abrasion cycles. Source data are provided as a Source Data file.

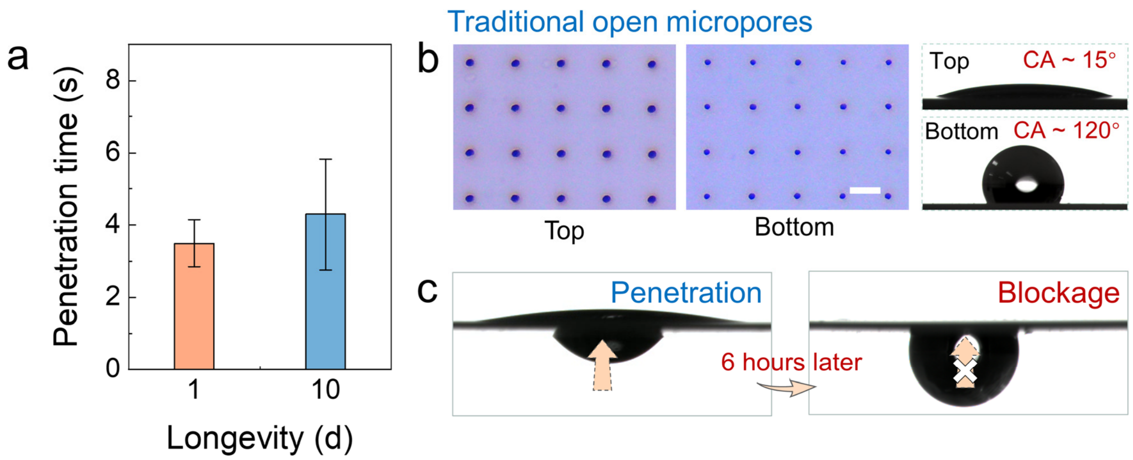

Fig. S12 a Water unidirectional penetration time of this robust Janus membrane exposed in air for 10 days in protection mode, **b** Optical microscopy images of the hydrophilic top and hydrophobic bottom sides of the traditional open micropore Janus membrane and the corresponding water contact angles. Scale bar: 100 μm. **c** Water unidirectional transportation function failure of traditional open polymer-based Janus membrane under only 6 hours of air exposure. The error bars represent the standard deviation of three independent measurements. Source data are provided as a Source Data file.

The thickness, micropore size and pore spacing of the control open micropore Janus membrane is 500 μm, 40 μm and 150 μm, respectively, same with the PG channels membranes in stretched state. Single-sided hydrophilic reagent treatment is also used to modify the top side of the control membrane to be hydrophilic with a CA of ~ 15°, and the bottom side exhibits intrinsic silicone hydrophobicity with a CA of ~ 120°.

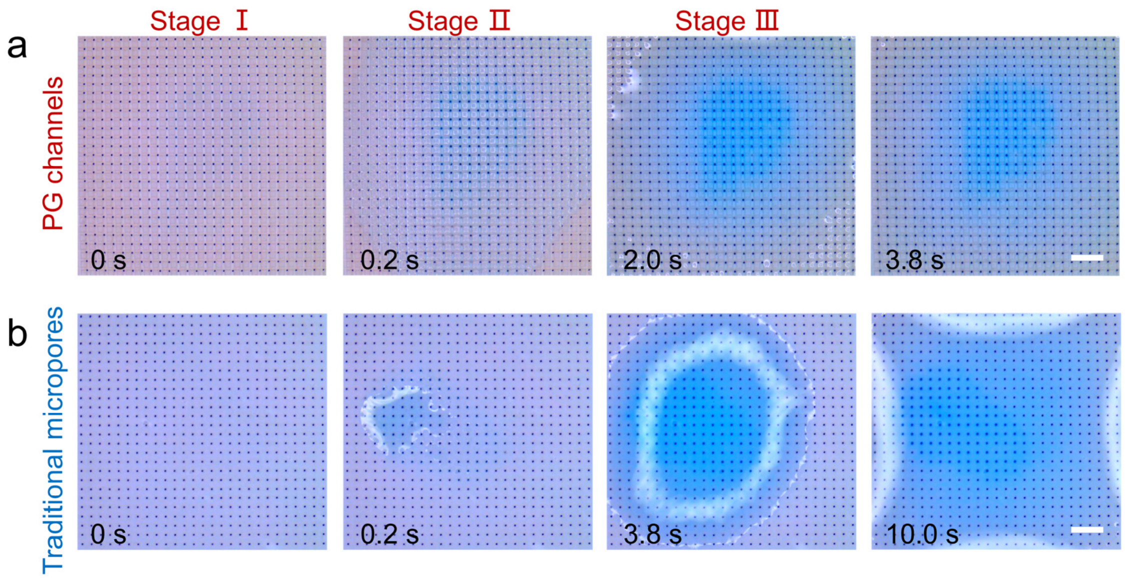

Fig. S13 Water spreading behaviors comparison on the Janus membrane top side of **a** PG channels and **b** traditional micropores. Scale bars: 500 μm.

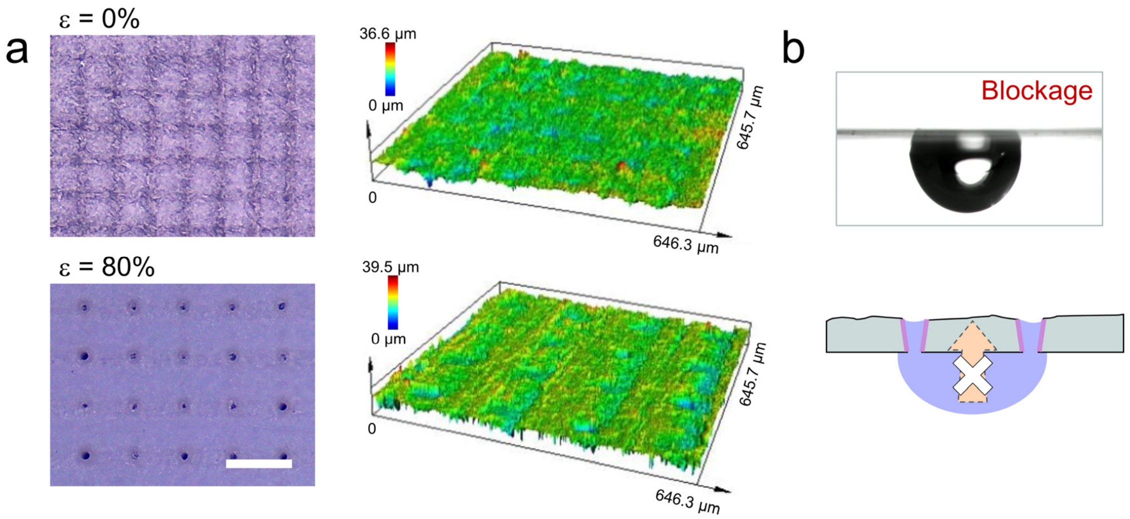

Fig. S14 a Optical and CLSM images of PG channels after 6000 abrasion cycles at stretching strains of 0% and 80%. Scale bars: 200 μm. **b** Optical image and schematic of a water droplet that cannot penetrate from the bottom to the top side.

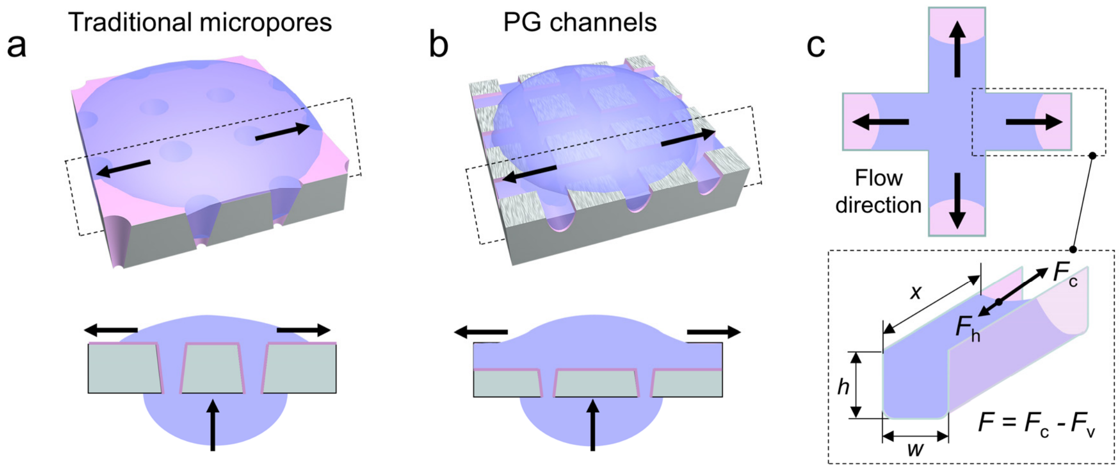

Fig. S15 Schematic diagrams of liquid spreading enhancement mechanism. Water spreading analysis comparison on Janus hydrophilic surface of **a** traditional micropores and **b** PG channels. **c** Water flow force analysis in microgrooves.

For the traditional Janus hydrophilic micropores surface, water spreading behavior occurs on a flat surface. In contrast, the PG channels have an obvious advantage in terms of accelerating water spreading. With the aid of force *F*, a liquid film quickly fills all the grid microgrooves, and the followed water spreading occurs on this preformed liquid film is much faster than that on a solid surface[7]. Consequently, the penetration time of water from the bottom to the top side is relatively decreased.

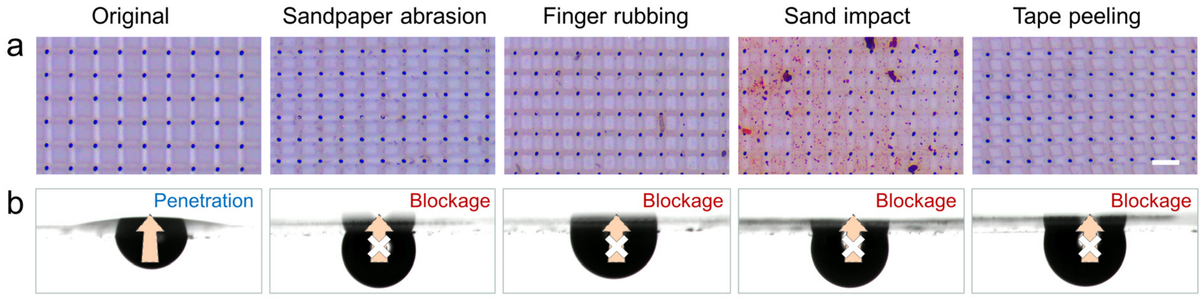

Fig. S16 **a** Top view photographs of PG channels and **b** corresponding water unidirectional penetration behaviors after the durability tests of sandpaper abrasion, finger rubbing, sand impact, and tape peeling, respectively. Scale bar: 200 μm.

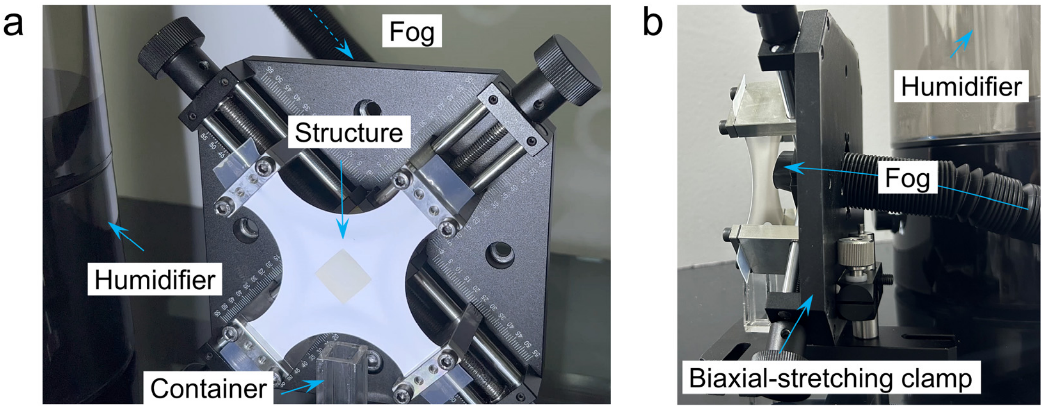

Fig. S17 a Front and **b** side view photograph of the fog-collection setup.

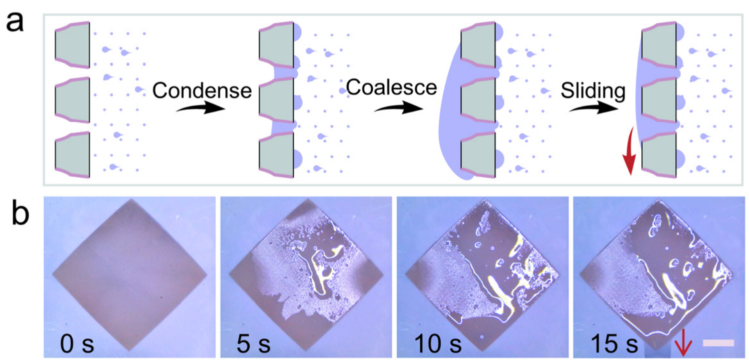

Fig. S18 a Schematic and **b** *in-situ* observation of fog condensation, water drop coalescence, and unidirectional sliding on the top PG channels surface before the durability tests. Scale bar: 5 mm.

References

1. Yang, D., Krasowska, M., Priest, C., Popescu, M. N. & Ralston, J. Dynamics of capillary-driven flow in open microchannels. *J. Phys. Chem. C* **115**, 18761-18769 (2011).
2. Rye, R. R., Yost, F. G. & O'Toole, E. J. Capillary flow in irregular surface grooves. *Langmuir* **14**, 3937-3943 (1998).
3. Li, Y. X. et al. Directional and adaptive oil self-transport on a multi-bioinspired grooved conical spine. *Adv. Funct. Mater.* **32**, 2201035 (2022).
4. Kolliopoulos, P., Jochem, K. S., Lade, R. K., Francis, L. F. & Kumar, S. Capillary flow with evaporation in open rectangular microchannels. *Langmuir* **35**, 8131-8143 (2019).
5. Ouali, F. F. et al. Wetting considerations in capillary rise and imbibition in closed square tubes and open rectangular cross-section channels. *Microfluid. Nanofluid.* **15**, 309-326 (2013).
6. Li, J., He, L., Liu, T., Cao, X. & Zhu, H. Preparation and characterization of PEG/SiO_2 composites as shape-stabilized phase change materials for thermal energy storage. *Sol. Energy Mater. Sol. Cells* **118**, 48-53 (2013).
7. Chen, H. et al. Ultrafast water harvesting and transport in hierarchical microchannels. *Nat. Mater.* **17**, 935-942 (2018).
