# Supplementary material for: Durable Janus membrane with on-demand mode switching fabricated by femtosecond laser
Source: Nat Commun. 2024 Feb 16;15:1443. doi: 10.1038/s41467-024-45926-4 (PMC10873403; doi:10.1038/s41467-024-45926-4)
Supplement: Supplementary file 3 — Description of Additional Supplementary Files [file 41467_2024_45926_MOESM3_ESM.pdf]

Description of Additional Supplementary Files

Supplementary Movie Legends:

Supplementary Movie 1: Water unidirectional transport behaviors of blockage in protection mode and penetration in Janus mode.

Supplementary Movie 2: Long-term mechanical abrasion test.

Supplementary Movie 3: Water anti-gravity unidirectional penetration behaviors before and after 2000 abrasion cycles.

Supplementary Movie 4: Water spreading behaviors comparison on the Janus hydrophilic surface of PG channels and traditional micropores.

Supplementary Movie 5: Water unidirectional penetration behavior in original state (before tests).

Supplementary Movie 6: Sandpaper abrasion test.

Supplementary Movie 7: Finger rubbing test.

Supplementary Movie 8: Sand impact test.

Supplementary Movie 9: Tape peeling test.

Supplementary Movie 10: Fog collection comparison before and after durability tests.
